# A rare *thyroid hormone receptor beta (THRβ)* gene mutation in a 15-year-old girl with thyroid hormone resistance syndrome: a case report

**DOI:** 10.1186/1752-1947-8-12

**Published:** 2014-01-06

**Authors:** Kawther El Shafie, Allal Ouhtit, Yousuf Al Farsi, Abeer Al Sayegh, Mohammed Al Shafaee

**Affiliations:** 1 Department of Family Medicine and Public Health, Sultan Qaboos University, PO Box 35, Postcode 123 Muscat, Sultanate Of Oman; 2 Department of Genetics, College of Medicine and Health Sciences, Sultan Qaboos University, Muscat, Sultanate of Oman

**Keywords:** Autosomal dominant, Consanguinity, Oman, *THRβ*, Thyroid hormone resistance

## Abstract

**Introduction:**

Resistance to thyroid hormone is a rare syndrome, where although the level of thyroid hormone is elevated, the level of thyroid stimulating hormone is not suppressed. The patient in our case report is, to the best of our knowledge, the first with this syndrome identified in Oman.

**Case presentation:**

In one Omani family, a 15-year-old girl of Arabian origin was pre-diagnosed with resistance to thyroid hormone. Blood sample was collected and deoxyribonucleic acid was isolated for molecular genetic testing. The results revealed a rare mutation A268G in the gene for *thyroid hormone receptor beta*. We believe that this mutation is the cause of the pathology in our patient.

**Conclusion:**

We report the presence of a rare mutation in the *thyroid hormone receptor beta* gene for the first time in the Omani population. Due to the rates of consanguinity being high among the Omani population, we are aiming to screen our patient’s family members and provide genetic counseling.

## Introduction

Resistance to thyroid hormone (RTH) is a rare disorder characterized by reduced tissue response to free thyroxine (FT_4_) hormone. RTH is an inherited disorder characterized by variable tissue hyposensitivity to 3,5,3′-l-triiodothyronine (T_3_), with persistent elevation of circulating free T_3_ (FT_3_) and FT_4_ levels, along with non-suppressed serum thyrotropin (TSH) [1,2]. Sporadic cases are common, but recessive cases are rare [1]. This syndrome was initially identified by Refetoff and colleagues, and since then 1000 cases have been identified from 339 families [2]. In 2008, Refetoff *et al.* reported more than 2000 individuals with RTH belonging to about 500 families [3]. RTH is associated with mutations in the *thyroid hormone receptor beta (THRβ)* gene; the *THRβ* protein is a nuclear hormone receptor for T_3_. Mutations in *THRβ* are associated with thyroid hormone resistance. The mode of inheritance can be autosomal dominant or recessive. The majority of patients are heterozygous for the mutant *THRβ* allele, consistent with the autosomal dominant pattern of inheritance [3]. Mutant *THRβs* can interfere with the function of the wild-type receptor in a dominant negative manner [4]. Lee *et al.* reported that at least 137 different *THRβ* mutations have been associated with RTH syndrome, affecting more than 40,000 people worldwide [5]. In our study, screening by direct sequencing analysis revealed a rare mutation (A268G) in the *THRβ* gene in a patient with RTH.

## Case presentation

A 15-year-old Omani girl of Arabian origin had presented with episodic palpitations since birth. At age 11 an electrocardiogram showed sinus tachycardia, whereas an echocardiogram was normal. Four years later, she presented with the episodic palpitations associated with mild dizziness. Her past medical history was normal and she reported no similar problem in any member of her family. A clinical examination showed a normal-looking girl with normal growth for her age. There was no evidence of mental retardation, but her parents reported that she had low performance at school. Her blood pressure was 124/59mmHg with a pulse rate of 108beats/min. She was not pale and her thyroid gland was not enlarged. A systemic examination was normal. Her electrocardiogram showed tachycardia with a few ventricular ectopics and an echocardiogram was once again normal.

A complete blood count was performed and her hemoglobin level was normal. Her C-reactive protein level was 97mg/L (normal range: 0 to 8mg/L), electrolytes were normal, potassium was 3.8mmol/L (normal range: 3.5 to 5.1mmol/L), sodium 139mol/L (normal range: 135 to 145mol/L), creatinine 33μmol/L (normal range: 29 to 56μmol/L), and urea 3.0mmol/L (normal range: 2.1 to 7.1mmol/L). Her FT_4_ level was 30.7pmol/L (normal range: 8.7 to 13.6pmol/L), FT3 9.0pmol/L (normal range: 3.8 to 6.0pmol/L), TSH 1.56mIU/L (normal range: 0.60 to 3.70mIU/L), thyroid antibodies (anti-thyroglobulin and antimicrosomal antibodies) 1IU/mL (normal range: 0 to 50IU/mL) and thyroglobulin 13μg/L (normal range: 3 to 30μg/L). Her thyroglobulin antibody level was <0.9IU/mL (normal range: 0.0 to 4.0IU/mL) and anti-thyroid receptor antibody level was 0.30UI/L (normal range: 0.00 to 1.50UI/l). A thyroid ultrasound showed altered echo texture with normal vascular flow on Doppler study. Tiny cystic areas of 2mm to 4mm were seen on both lobes. There were no significant nodules in her thyroid gland, and no lymphadenopathy was detected in her neck. A technetium-99m thyroid scan showed a total uptake of 3.2% (normal uptake: 1% to 4%) seen on both lobes, and no cold nodules were observed. Her prolactin level was 727mIU/L (normal range: 71 to 566mIU/L). Magnetic resonance imaging scan of the pituitary gland and brain showed that both her pituitary gland and brain were normal in size and morphology.

Blood samples were collected from our patient and her deoxyribonucleic acid (DNA) was isolated. The samples were sent to an accredited laboratory in the Department of Clinical Genetics at the University of Amsterdam, Netherlands. Direct sequencing analysis of the full *THRβ* gene was performed. The results revealed the presence of heterozygous mutation c.803C > G in *THRβ*. This nucleotide change results in the amino acid change p.Ala268Gly in *THRβ*. Hence, this mutation indicates that our patient was most likely affected with RTH. We therefore treated her with beta-blockers to control her tachycardia.

## Discussion

RTH is characterized by tissue hyposensitivity to elevated levels of FT_3_ and FT_4_ in association with unsuppressed serum thyrotropin (TSH) [6]. Patients with congenital RTH can be clinically euthyroid, but often show increased levels of FT_4_ and FT_3_ with unsuppressed TSH [7]. In our patient, the elevated levels of thyroid hormones (FT_3_ and FT_4_) with normal levels of TSH, exclusion of a pituitary tumor by magnetic resonance imaging and the presence of a rare mutation, p.Ala268Gly, in *THRβ* support the diagnosis of RTH. The DNA analysis of our patient showed the presence of a heterozygous mutation c.803C > G in *THRβ*. An alpha subunit to TSH ratio, which would give more support to the diagnosis, will be calculated in our patient during the screening of her family. Rivolta *et al.* reported that 85% of patients were heterozygous for the mutant *THRβ* allele, consistent with the autosomal dominant mode of inheritance [6]. In our patient, the c.803C > G nucleotide change, resulting in the amino acid change p.A268G, altered the highly conserved amino acid *THRβ*. This mutation was not detected in 60 other index patients and 13,000 control alleles in the indexed laboratory. This rare mutation has been previously described only in a four-generation family with RTH [8] and in one South American patient [6].

The incidence of RTH is one in 40,000 [9]. The clinical presentation of this syndrome is variable. Some individuals tend to be normal and the syndrome is detected only on routine investigations, whereas others may display features such as goiter, hyper- and hypothyroidism, delayed bone maturation, short stature and attention deficit hyperactive disorder [10]. Goiter is the most common finding, reported in 66% to 95% of patients [11]. Our patient had no goiter. According to the clinical presentation, this syndrome has been divided into two entities; generalized type (GRTH) and pituitary type (PRTH). Patients with RTH who appear normal or hypothyroid are considered to have GRTH, and those with thyrotoxic clinical presentation are considered to have PRTH [12]. Accordingly, we would classify our patient as having PRTH, because of her sinus tachycardia. One of the most important reasons for recognizing RTH is that its management is different from other types of thyroid dysfunction. Also, it is important to distinguish GRTH from PRTH using clinical criteria as the management of these two types differ. Patients with PRTH should not be treated with anti-thyroid drugs, as any reduction of the thyroid hormone will aggravate the symptoms, reduce growth and increase the goiter size. Patients with this type, similar to our patient, are usually the most difficult to treat. Patients with GRTH can benefit from thyroxin administration, since it reduces TSH level and the goiter [5].

The alpha subunit of the TSH (α-GSU/TSH) was not measured in our patient, which is considered as a limitation of this case report, because of non-availability of the test. While the main difference between patients with TSH-producing pituitary tumors (TSHomas) and RTH is the presence of symptoms of hyperthyroidism; a minority of patients with RTH might display signs of hyperthyroidism involving some organs and not others, known as PRTH [13]. Nevertheless, the differential diagnosis between these forms of RTH and TSHomas may be difficult, and elevated α-GSU concentrations or high α-GSU/TSH molar ratio, favors TSHoma. More interestingly, similar biochemical data in relatives favors RTH. Therefore, α-GSU concentrations or high α-GSU/TSH molar ratio will be measured in the next study, which will include our patient and other members of her family.

## Conclusion

We report the presence of a rare mutation in the *THRβ* gene for what we believe to be the first time in the Omani population. Because of high rates of consanguinity in this population, screening of a large number of our patient’s family members is required to identify similar or different types of gene mutations. On the basis of the result, appropriate treatment related to the type of clinical presentations can be provided to the relatives. Futhermore, genetic counseling can also be provided to those who intend to have consanguineous unions and can help in prevention of the disease.

## Consent

Written informed consent was obtained from the patient’s guardian for publication of this case report. A copy of the written consent is available for review by the Editor-in-Chief of this journal.

## Abbreviations

FT3: Free triiodothyroinine; FT4: Free thyroxin; GRTH: Generalized thyroid hormone resistance; PRTH: Pituitary thyroid hormone resistance; RTH: Thyroid hormone resistance; T3: 3,5,3′-l-triiodothyronine; T4: Thyroxine; TSH: Thyroid stimulating hormone or Thyrotropin; THRβ: *Thyroid hormone receptor beta.*

## Competing interests

The authors declare that they have no competing interests.

## Authors’ contributions

KE: conceived of the study, and participated in its design and coordination and helped to draft the manuscript; AO: carried out the molecular genetic studies literature, participated in the coordination and helped to draft the manuscript. YA: participated in the design of the study and contributed to the interpretation of the results. AA: carried out and interpreted the results of the molecular genetic tests. MA: conceived of the study, participated in its design, and contributed to drafting the manuscript. All authors read and approved the final manuscript.
